# Eye, Ear, Nose and Throat

**Published:** 1893-07

**Authors:** 


### EYE, EAR, NOSE AND THROAT.

EDITED BY DR. ARTHUR G. HOBBS, ASSISTED BY
DR. GEORGE BROWN.

### EYE-PARALYSES.

The *American Journal of Ophthalmology* (December, 1892) contains a very comprehensive article on the paralyses of ocular muscles. The following is a summary:

1. All cases of lateral conjugate paralysis are of central origin.

2. When the paralysis is on the same side as other paralyses, the lesion is on the opposite side of the brain. Such paralyses as a rule are transitory, and follow almost any sudden lesion, and often only show themselves as a prevailing position of the eye, and not as a true paralysis, or even paresis.

3. When the paralysis is crossed with the paralyses below, the lesion is in the pons-medulla region.

The above three conclusions are equally true of spasms.

4. A gradual development of conjugate paralysis clearly points to the region of the sixth nucleus of the same side as affected.

5. Paraylsis of up or down motions, or both motions, indicate disease in the region of the corpora quadrigemina, at the point of exit.

6. Reasoning from analogy, paralysis of convergence points to disease in the central gray below the aqueduct, but as yet autopsies are lacking.

7. Picked paralysis of parts of a third nerve strongly suggests central disease, but is not proof of it.

8. A majority of cases of eye-paralysis occur in the syphilitic.

9. A paralysis which changes rapidly, quickly showing fatigue, is probably central in origin.

10. Transitory paralysis in the syphilitic is strongly suggestive of future tabes.

11. An eye paralysis, however simple it may seem, is always a just cause for suspicion of trouble to come, and demands a prompt and thorough examination of the patient.

12. There is no evidence that there is any form of connection between the sixth nucleus and the third except in the cerebrum.—*The Cincinnati Lancet-Clinic.*

---

### PAPILLOMA OF THE LARYNX.

Dr. T. Payson Clarke says in *The Boston Medical and Surgical Journal:*

What is the best method of treating these growths? Is it possible to prevent their recurrence? In the first place it must be borne in mind that these are benign growths, only dangerous as they offer an obstruction to respiration by their increasing size, which is in adults always sufficiently slow to give ample warning of danger. It must also be remembered that their natural tendency has been shown to be, sooner or later, to disappear. Our first aim therefore must be to choose some method of operation which cannot injure in any way the delicate structures of the larnyx. Such a method, it seems to me, is offered by the use of laryngeal forceps, especially Schrotter's tube forceps. In operating accuracy in bringing the instrument quickly to the desired spot in the larynx is an all-important factor. Undue haste to grasp the growth and remove it must be carefully avoided. The operator must see distinctly what he is about to grasp with his forceps. To quote Dr. Hooper: "The peculiar nature of these benign papillary growths and the delicacy of the laryngeal structures demand that when surgical interference is necessary it should be conducted with the greatest prudence and patience."

The application of one of the various caustics to the part from which the growth has been torn seems to be either useless or harmful, and sometimes even dangerous, to life. In some cases the use of caustics or the galvano-cautery has only stimulated the growth, while, on the other hand, cases are on record where from the use of powerful caustics adhesions have been formed which have permanently impaired the voice.

In treatment, therefore, it is well to avoid agents such as powerful caustics, which may do injury to the normal structures of the larynx, and simply to remove as thoroughly as possible by other means whenever it becomes of sufficient size to interfere with phonation or respiration, bearing in mind its benign character and its tendency to ultimate atrophy and disappearance.

---

### CATHETERIZATION OF THE EUSTACHIAN TUBE.

Loewemberg, of Paris, describes a new method of catheterizing the Eustachian tube. The principal difficulties during the first stage of the operation are the horizontal deviations of the septum. During the second stage, when the point of the instrument leaves the nasal cavity, it is difficult to tell when we are in front of the pharyngeal orifice of the Eustachian tube. When Loewemberg thinks his catheter has arrived at the end of the post-nasal cavity, he orders the patient to execute a movement of swallowing. If the instrument has not yet passed the nasal cavity, the hand holding the instrument feels no shock; but if the instrument has passed into the pharyngeal cavity, the soft palate, when contracting, pushes it sometimes into the tube, or, at least, displaces it, which movement is seen and felt by the hand. The right distance once found, it will be useful to mark it on the instrument.—*Gaceta médica de México.*—*The Universal Medical Journal, April,* 1893.

---

### COCAINE POISONING.

Dr. A. R. Baker, of Cleveland, in a paper read before the N. E. Ohio Union Medical Association, reported a case of cocaine poisoning with very alarming symptoms, produced by eight drops of a 6 per cent. solution in the eye. Ten fatal cases were also read, indicating that a fatal dose is much smaller than generally thought, especially if the drug be used about the mucous membranes of the mouth, nose, or throat, or by hypodermic injection.—*Western Druggist.*

---

### OTITIS MEDIA.

Dr. Hinton recommends:

℞. Liq. plumb. subac., *m*xx.
Acid. acetic. dil., *m*vj.
Liq. opii sedativ., *m*xx.
Aq. dest., q. s. ad ℥j.

M. Sig.: Drop 10 drops, warm, into the ear.

—*The Medical Bulletin.*

